# Western Lifestyle and Increased Prevalence of Atopic Diseases: *An Example from a Small Papua New Guinean Island*

**DOI:** 10.1097/WOX.0b013e3181accf27

**Published:** 2009-07-15

**Authors:** Oliver CH Herbert, Ross STC Barnetson, Wolfgang Weninger, Ursula Krämer, Heidrun Behrendt, Johannes Ring

**Affiliations:** 1 Division of Environmental Dermatology and Allergology, Department of Dermatology and Allergy, Technical University Munich, Biedersteinerstrasse 29, 80802 Munich, Germany; 2 Department of Dermatology, Royal Prince Alfred Hospital, Sydney, Australia; 3 Institute for Environmental Medical Research, Heinrich Heine University, Düsseldorf, Germany; 4 The Menzies Centre for Health Policy, University of Sydney, Sydney, Australia

**Keywords:** allergy prevalence, atopic disease, modern versus traditional lifestyle, Westernization, infections, parasites, public health, tropics, Papua New Guinea, Karkar Island

## Abstract

**Background:**

Allergic diseases represent an increasing problem in public health in most modern societies as their prevalence has risen markedly during recent decades. Nevertheless, the causes of this increase are not yet fully explained.

**Objective:**

We investigated the correlation of Western lifestyle pattern in varying intensity to the prevalence of atopic diseases in 5 small villages on Karkar Island, in northeast Papua New Guinea.

**Methods:**

Two hundred forty-eight native people from 5 villages on tropical Karkar Island have been included in this study. The degree of Western lifestyle was assessed (questionnaire and observation) for each village. The prevalence of atopic diseases was evaluated by personal and family history, physical and dermatological examination, skin prick test (10 allergens), and measurement of total and specific immunoglobulin E levels (20 common allergens).

**Results:**

The more easily accessible and thus more "modern" and westernized coastal villages showed a significantly higher prevalence of habitants suffering from atopic diseases than a traditional mountain village (6.8% vs 0.0%, *P *= 0.034, Fisher exact test). A total of 4.4% (11/248) of the examined islanders suffered from an atopic disease. Atopic eczema seems to be absent on Karkar Island.

**Conclusions:**

The results of this study suggest that so-called Western lifestyle may contribute to the development of atopic diseases.

## Introduction

Studies from industrialized countries all over the world indicate an increasing prevalence of allergic diseases and atopy during recent decades [1-8]. Allergies are becoming a more and more serious problem in most modern societies. As the increase in the prevalence of allergies has occurred during a period of rapid technological progress, it is plausible that the modern Western lifestyle might be involved in this increase [9,10]. Therefore, this study focused on 2 main questions:

1. What is the prevalence of atopic diseases in the absence of typical Western lifestyle?

2. Is there a higher prevalence of atopic diseases in traditional societies when aspects of Western lifestyle have already been introduced to a certain extent?

**Figure 1:**
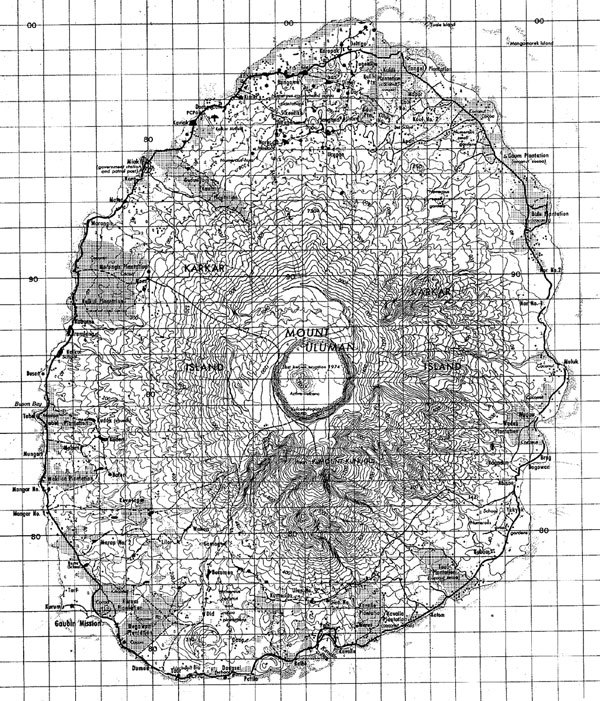
**Karkar Island (370 km^2^)**.

Karkar Island (Figure 1), a small tropical volcanic island (370 km^2^)[11] on the northeast shore of Papua New Guinea, provided an ideal place for studying this problem. So far, there is no industry on the island and individual car traffic is negligible. The only sources of outdoor anthropogenic air pollution are cooking fires. Most people live on subsistence gardening, fishing, or working on coconut plantations.

**Figure 2:**
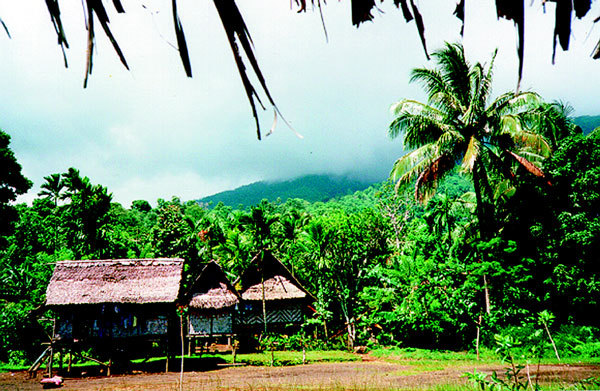
**Traditional mountain village**.

The 62,000 habitants live in roughly 2 types of villages: the coastal and the mountain villages. Because of a coastal road, the coastal villages are easily accessible, whereas the mountain villages on the slopes of the 1833-m-high volcano (Mount Kunugui) are rather isolated (Figure 2). From the beginning of the 20th century to the present time, Western influence has predominantly affected the coastal areas where the wharves and the airstrip are situated. For instance, the Gaubin Mission Hospital, founded in 1948 [12] and representing the Western medical system, and most of the trade stores selling Western imported goods can be found on the coastal road.

We included 5 villages with a total of 248 islanders in this survey: Gaubin, Kurum, and Kavailo, representing the more modern coastal villages, and Gamog, representing a traditional mountain village, a 4-hour walk from the shore. The village Did is in an intermediate position, as it is situated a little further inland, between the 3 coastal villages and Gamog (2-hour walk from the shore).

## Methods

### Study Population

The study was conducted on Karkar Island, Papua New Guinea. The inhabitants are divided into 2 linguistic groups: The Takia in the South and the Waskia in the North of the island. Data were collected in 1996/97 and 2001 in 5 different Takia villages: Gaubin, Kurum, and Kavailo on the Coast; Did a little further inland; and Gamog on the slopes of the Mount Kunugui volcano.

Considering the total number of inhabitants in the 5 villages, a representative percentage of persons have been included in the study: 31.0% (26/84) of the population of Gaubin, 8.1% (62/765) of Kurum, 17.3% (60/347) of Kavailo, 8.0% (42/525) of Did, and 16.2% (58/358) of Gamog. Altogether 248 randomly selected volunteers took part in the study (convenient sample). They were approached directly in their home villages. Resources for the study were limited, yet in all villages most people were keen on participating--despite skin tests and blood sampling. Only the first people who appeared after being in-formed by traditional acoustical means (Garamut wood drum) could be included in the study. The average age was 25.7 (± 15.2) years; 9% (23/246) of the subjects were children under the age of 10. The percentage of women was 53% (132/248) and that of men 47% (116/248). The following data were obtained on each of the participants.

### General Medical and Allergy History

A detailed medical history consisting of 29 questions and focusing on atopic diathesis, personal and family history of atopic diseases, general health, utilization of Western drugs, and general life conditions (standardized, self-developed questionnaire, validated in 30 patients with atopic disease) was obtained. The answers were translated from Neo-Melanesian Tok-Pidgin to German and English.

### Skin Prick Tests

Skin prick tests (SPT) were performed at the volar side of the lower forearm of the participants and the wheal diameters were read after 20 minutes. Histamine and 0.9% sodium chloride were used as controls. Seven aeroallergens (*Artemisia vulgaris*, grass-pollen mixture, *Dermatophagoides pteronyssinus, Dermatophagoides farinae*, dog epithelium, cat epithelium, and rat epithelium) and 3 food allergens (hen's egg, codfish, and cow's milk) were tested (Allergopharma, Reinbeck, Germany). Additionally, we included SPT results against cockroach collected in 2001-2002. Wheal diameters of more than 3 mm were considered positive.

### Total and Specific Immunoglobulin E

The evaluation of medical histories and the SPT were followed by blood sampling directly in the villages. Within a maximum time of 4 hours--depending on the location of the village--the samples were transported to Gaubin Hospital in a cooled container. In the laboratory of the hospital the blood was centrifuged and sera were stored frozen until total and specific immunoglobulin E (IgE) measurements were performed at the Department of Dermatology and Allergy Biederstein, Technical University München, Munich, Germany. The Pharmacia CAP System IgE fluorescent enzyme immunoassay was used. Specific IgE to 20 different allergens was evaluated quantitatively with the Pharmacia CAP System radioallergosorbent test (RAST) fluorescent enzyme immunoassay. The results are given in RAST classes from 1 to 6. The following 20 allergens were measured: *D. pteronyssinus, D. farinae*, dog epithelium, cat epithelium, rat epithelium, pig epithelium, egg protein, cow's milk protein, codfish (*Cadus morua *), sweet potato (*Ipomea batatas*), bastard mackerel (*Trachurus japonicus*), mango (*Mangifera indica*), banana (*Musa sapientum*/*paradisiaca*), papaya (*Carica papaya*), arabic gum, timothy grass (*Phleum pratense*), mugwort (*Artemisia vulgaris*), cockroach (*Blattella germanica*), latex (*Hevea brasiliensis*), and *Ascaris lumbricoides*.

### Dermatological Examination

The medical examination focused on clinical signs of atopic airway diseases (such as rhinoconjunctivitis, rhinitis, and dyspnea) and skin manifestations of allergy or atopy. The frequency and extent of symptoms and the correlation of the medical history of clinical signs and allergen exposure were recorded and stigmata of atopy were registered. We considered that a person was suffering from an atopic disease by combining the allergy history with SPT and RAST results.

### Statistical Analyses

The following analytical statistical tests were applied: as a parametric test, the Student *t *test; as a nonparametric test, the Wilcoxon rank sum test; and for noninterval data, the *χ*^2 ^test/Fisher exact test. All medical histories, translations from Neo-Melanesian Tok-Pidgin, SPT, and total and specific IgE measurements were conducted by the same examiner.

## Results

### 1. General Lifestyle and Health Conditions on Karkar Island

**Figure 3:**
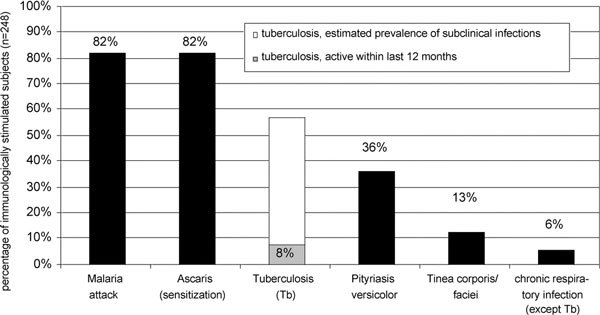
**Prevalence of different kinds of infections on Karkar Island**. The parasitic infections with Plasmodia and Ascaris play a preeminent role. Bacteriological and fungal infections are extremely common as well.

Many aspects of daily life show that the lifestyle on Karkar Island is still a traditional one. Eighty-three percent of the investigated adult subjects (169/203) lived on subsistence gardening. The others were fishermen or worked as teachers, as pastors, as storekeepers, in health service, or on coconut plantations. Adults had an average of 3.2 (± 2.6) children. Only 3% (7/224) of the investigated subjects had no siblings; the average number of siblings was 4.8 (± 2.5) and 39% (88/224) had 6 or more siblings. Parasitic, bacteriological, viral, and fungal infections play a predominant role in this tropical environment. Eighty-two percent of the subjects (173/211) reported a malaria attack within the previous 12 months. Antibodies against *A. lumbricoides *could be detected in 82% (203/248) of the investigated blood samples. Eight percent stated that they had been treated for active tuberculosis within the last 12 months. At the time of investigation, 36% (89/247) of the subjects suffered from pityriasis versicolor and 13% (32/247) from tinea corporis and/or faciei. A chronic respiratory infection (except tuberculosis) within the last 12 months was reported by 6% (15/248) of the study participants. Figure 3 highlights the relevance of the abovementioned infections on Karkar Island.

**Figure 4:**
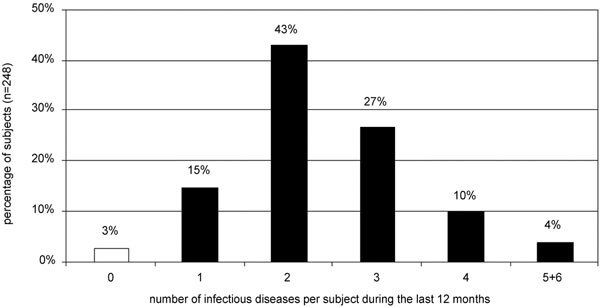
**Percentage of islanders suffering from 0 to 6 (sub)acute infections**. Malaria (attack within last 12 months), Ascaris infestation (present antibodies, presumably reflecting acute infestation), tuberculosis (active within last 12 months), pityriasis versicolor (present), tinea corporis and/or faciei (present), and respiratory tract infections except tuberculosis (within last 12 months) have been considered.

The immune system of 82% of the islanders (204/248) had to cope with more than just 1 of the above-mentioned 6 infections at the same time. Concerning tuberculosis only, the cases of active disease within the last 12 months were associated with definite individuals and thus have been taken into account. Only 3% of the subjects (7/248) had no infection. The detailed numbers of infections per subject are shown in Figure 4.

### 2. Traditional Lifestyle in the Mountain Village Compared with the Coastal Villages

Although the general lifestyle on Karkar Island is a traditional one, differences between the remote mountain village Gamog and the coastal villages are conspicuous. In Gamog significantly more people had a history of dermatophyte infection (Gamog, 28%; coastal villages, 12%; *P *< 0.01, *χ *^2 ^test). A significantly higher percentage of Gamog villagers reported worms in their stool (Gamog, 33%; coastal villages, 13%; *P *< 0.01, *χ *^2 ^test). Significantly more people in Gamog had a medical history of a severe respiratory tract infection (except tuberculosis) in the year of the inquiry (Gamog, 11%; coastal villages, 3%; *P *< 0.05, Fisher exact test). In Gamog a significantly higher percentage of individuals suffered from chronic back pain than those in the coastal villages (Gamog, 11%; coastal villages, 1%; *P *< 0.005, Fisher exact test). This finding is most likely related to the examiner's observation of higher physical strain in the mountain villages. Significantly more villagers in Gamog kept a dog and/or cat (Gamog, 70%; coastal villages, 53%; *P *< 0.05, *χ *^2 ^test). Table 1 highlights the significant differences of lifestyle variables between coastal and mountain villages. Furthermore, in Gamog there are no electrical generators (on the coast: a few generators in every village), no cars (coast: very few cars), no asphalt streets (coast: just in recent years some streets have been asphalted), no chemicals (coast: some chemicals, for example, detergents, are available in small shops situated in the coastal area), less use of industrially produced foods (coast: increasing use of industrially produced food, for example, white flour, white sugar, white rice, biscuits, and tinned meat or fish.), no television (coast: very few), and less influence of Western medicine (coast: Gaubin Mission Hospital since 1948).

**Table 1:** Infections and Lifestyle Variables in Coastal and Mountain Villages: Significant Differences

Parameter	Coast	Mountain	*P*
Dermatophyte infection	12% (16/133)	28% (13/46)	*P *< 0.01
Helminth infestation	13% (17/127)	33% (15/46)	*P *< 0.01
Respiratory tract infection	3% (4/133)	11% (5/46)	*P *< 0.05
Chronic back pain	1% (1/133)	11% (5/46)	*P *< 0.005
Pet keeping	53% (70/133)	70% (32/46)	*P *< 0.05

No significant differences were found concerning tuberculosis, malaria, ear infections, smoking habits, and the use of betel nut.

No significant differences between mountain and coastal villages were found concerning the following parts of the medical histories: prevalence of atopic diseases in family history, smoking habits, use of betel nut, tuberculosis, malaria, and ear infections. There was no significant overall difference with respect to total IgE, SPT results, and specific IgE against aeroallergens (Herbert et al, in preparation).

### 3. Low Prevalence of Atopic Diseases on Karkar Island

The overall prevalence of atopic diseases in the 5 investigated villages on Karkar Island was 4.4%. Thus, only 11 of the 248 examined islanders suffered from rhinoconjunctivitis and/or asthma. No atopic eczema was observed and there were no medical histories of signs of atopic eczema either. Stigmata of atopy were difficult to evaluate and showed no significant differences between areas.

### 4. Skin Prick Test and Specific IgE Results

The 3 most common aeroallergen sensitizations were the following: 16.4% of the villagers (40/244) showed a positive SPT against *D. pteronyssinus*, 15.4% (22/143) against cockroach, and 1.2% (3/243) against grass-pollen mixture. This order was reflected by the RAST results. As already mentioned, anti-Ascaris IgE was present in 82% (203/248) of the blood samples.

### 5. Prevalence of Atopic Diseases in the Mountain Village Compared with the Coastal Villages

**Figure 5:**
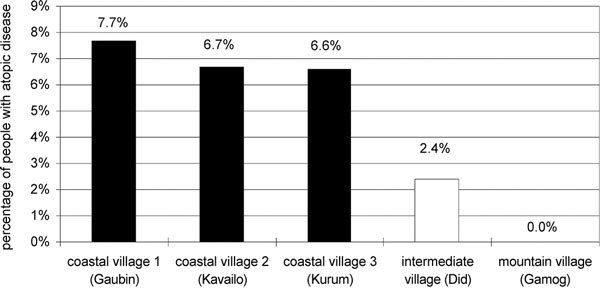
**Percentage of people suffering from atopic disease in the coastal villages Gaubin, Kavailo, and Kurum (black columns), the intermediate village Did (light column), and the mountain village Gamog**. The difference between coastal and mountain villages was significant (*P *< 0.05, Fisher exact test).

No atopic disease was found in the isolated mountain village Gamog. Yet the prevalence of atopic diseases in the coastal villages Gaubin, Kavailo, and Kurum was 6.8% (10/148). This difference in prevalence of atopic diseases between traditional mountain villages and more modern coastal villages was significant (*P *< 0.05, Fisher exact test). The village Did, located between the 3 coastal villages and Gamog, took an intermediate position with an atopic diseases prevalence of 2.4% (1/42), which was higher than that in Gamog, but still lower compared with that of the coastal villages. Nevertheless, these differences were not significant (Figure 5).

With a prevalence of 3.2% (8/248) rhinitis/rhinoconjunctivitis was the most common manifestation of an atopic disease. In the coastal villages, 5.4% (8/148) of the subjects suffered from rhinitis/rhinoconjunctivitis, but in the interme-diate village and in the mountain village, 0% of the subjects suffered from rhinitis/rhinoconjunctivitis (0/42 and 0/58 respectively). Thus, in the coastal villages, rhinitis/rhinocon-junctivitis was significantly more common than in the non-coastal villages (mountain and intermediate villages) (*P *= 0.015, Fisher exact test). The prevalence of allergic asthma on Karkar Island was 2.4% (6/248). Like rhinitis/rhinoconjunctivitis also allergic asthma was most common in the coastal villages (3.4%). In the intermediate village only 2.4% (1/42) suffered from allergic asthma and in the mountain village no subjects suffered from allergic asthma. Yet these differences were not significant (Fisher exact test). Atopic eczema was absent in all 5 villages (Table 2).

**Table 2:** Prevalence of Atopic Manifestations; Rhinitis/Rhinoconjunctivitis, Allergic Asthma, and Atopic Eczema in the Coastal, Intermediate, and Mountain Villages

	Rhinitis/Rhinoconjunctivits	Allergic Asthma	Atopic Eczema	Atopic Disease (Total)
Coastal villages	5.4% (8/148)	3.4% (5/148)	0.0% (0/148)	6.8% (10/148)
Intermediate village	0.0% (0/42)	2.4% (1/42)	0.0% (0/42)	2.4% (1/42)
Mountain village	0.0% (0/58)	0.0% (0/58)	0.0% (0/58)	0.0% (0/58)
All villages	3.2% (8/248)	2.4% (6/248)	0.0% (0/248)	4.4% (11/248)

### 6. Significant Differences between Subjects with and without Atopic Diseases

In the group of islanders suffering from an atopic disease, 25% of the people worked in typical Western occupations, whereas in the group of islanders without an atopic disease only 12.7% did. Yet this difference was not significant (Wilcoxon test). Western occupations include the following: employees of the Western health system, teachers, pastors, and shopkeepers. Traditional occupations are as follows: farmers, workers in coconut plantations, fishermen, and persons without a specific occupation.

Furthermore, people suffering from atopic disease took significantly (*P *< 0.05) more acetylsalicylic acid than people without atopic disease (Wilcoxon test). Villagers with atopic disease used acetylsalicylic acid 20.4 times a year, whereas people without atopic disease stated to take this drug just 12.0 times a year (arithmetic mean). Antibiotics (eg, amoxicillin and chloramphenicol) were used in a similar pattern by subjects with and without atopic disease and by subjects in coastal and mountain villages.

## Discussion

The results of this study suggest that a traditional lifestyle may protect against atopic diseases. Yet 1 major limitation of this investigation is the rather small sample size. Nevertheless, the selected villages were quite representative for the population on Karkar Island. A multivariate analysis was not performed.

### 1. The Traditional Lifestyle on Karkar Island

As 83% of the adults lived on subsistence gardening, lack of physical exercise--so typical for industrialized countries [13]--is not a problem on Karkar Island.

Except for cooking fires, there is no other source of air pollution. It has been suggested that within large families like those on Karkar Island the probability of developing an atopic disease would be decreased [14]. Traditionally, children are breast-fed over long periods of time and the use of milk powder just started to spread over the island slowly since approximately 2000. Breast-feeding has been shown to decrease the development of atopic diseases [15]. Traditions generally retain an important role, including the importance of the clan structure, the celebration of traditional feasts, the chewing of betel nut, the use of traditional herbal medicine, and--despite evangelization--the broad belief in magic and sorcery (Herbert, Dietrich Reimer Verlag in print, English edition in preparation).

Among the many aspects of traditional life, which may stimulate the immune system of the islanders, infections play a predominant role. The area is highly endemic for plasmodium falciparum, vivax, and ovale. Therefore, people develop a partial immunity to these protozoa. Although malaria sometimes can be life threatening (especially for children), symptoms are mostly only moderate and may be misinterpreted as a cold. Thus, a malaria attack within the last 12 months might have affected even more than 82% of the islanders. The broad IgE sensitization against *A. lumbricoides *seems to widely reflect current helminth infestations. Stool samples from the Gaubin Hospital laboratory and data from an investigation carried out in Kevasob village on Karkar Island [16] support this suggestion. Furthermore, the prevalence of hookworm infestation (*Ancylostoma duodenale*/*americanus*) is even higher than the prevalence of infestation with *A. lumbricoides*. Infections with *Strongyloides stercoralis *are found as well. Considering these facts, most of the islanders will have suffered from 1 or even several nematode infestations within the last 12 months. Helminths are known to exert a substantial influence on the host's immune response [17] and results of an investigation by Hagel et al [18] carried out on Venezuelan children suggest that the formation of specific anti-Ascaris IgE reflects an effective immune response to these parasites.

In addition to parasitic infestations, bacteriological infections are also extremely common. Pyodermas of different kinds, and the already mentioned chronic respiratory infections, were observed in many islanders. When the percentage of islanders whose immune system was actually confronted with mycobacteria is being estimated, it has to be taken into account that the manifestation index of tuberculosis is only under 10% [19]. Thus, more than 90% of tuberculosis infections entail a clinically unapparent immune stimulation (subclinical infection). Chronic dermatophyte and yeast (Pityriasis versicolor) infections are immunomodulatory as well [20].

The following numbers give the best idea of the eminent immune-stimulating role of infections on Karkar Island. Just 3% of the subjects (7/248) had none of the abovementioned 6 infections. Furthermore, we have to take into account the broad variety of other common infections like pneumonia, viral hepatitis, frambesia tropica (Yaws), pyodermas, mastitis puerperalis, scabies, urogenital tract infections, gonorrhea, pyelonephritis, tooth problems, and gastritis. Thus, we conclude there is not a single islander not suffering from 1 or more infections.

### 2. Low Prevalence of Atopic Diseases on Karkar Island

The low overall prevalence of atopic disease on Karkar Island (4.4%) cannot be explained by a lower allergen load. The tropical environmental conditions on the island are likely to entail a high load of various natural antigens, which are often the cause of allergic diseases in Western countries. House dust mite, cockroach, animal epithelia, and mold allergens may be prevalent. This assumption is supported by the RAST results. Most of the subjects showed specific IgE antibodies against environmental antigens. The traditional lifestyle seems to protect the Karkar Islanders from developing atopic diseases--interestingly, despite ongoing specific IgE production. One possible explanation would be that the traditional lifestyle lowers the impact of IgE on effector cells (eg, mast cells and basophils).

### 3. Traditional Lifestyle and Low Prevalence of Atopic Diseases in the Mountain Village Compared with the Coastal Villages

The hypothesis that a traditional lifestyle protects against atopic diseases is further supported by the coincidence of the more modern lifestyle of coastal villagers and their significantly increased risk of developing atopic diseases. Some aspects of a more traditional way of life in the remote mountain village Gamog may be repeated in short: In Gamog there are no electricity, no cars, no asphalt streets, no chemicals, almost no use of industrially produced foods, and less influence of Western medicine. The geographical and partial social isolation of the mountain village Gamog and the retention of traditional gardening practices has already been described by King et al. [21]. The significantly increased number of mountain villagers keeping pets can also be regarded as an indicator of a traditional lifestyle. Considering all lifestyle variables, differences between villages were most striking in the field of infections: the number of dermatophyte infections, worm infestations, and severe respiratory tract infections were significantly higher in the mountain village compared to those in the coastal villages.

The more traditional lifestyle in the isolated mountain village was correlated with a significantly lower prevalence of atopic diseases: actually, no atopic disease at all was found in the mountain village where typical aspects of modern Western lifestyle were absent. Yet in the 3 easily accessible and more modern coastal villages, the prevalence of atopic diseases (rhinitis/rhinoconjunctivitis and asthma) was 6.8% (10/148). As in Western countries, the most common mani-festation of an atopic disease on Karkar Island was rhinitis/rhinoconjunctivitis.

Another finding is that in a period of 22 years there was a significant increase (*P *< 0.05) in the reported asthma prevalence (extrinsic and intrinsic asthma) in the coastal villages: from 1.5% in 1974 [22] up to 4.7% in 1996. In the traditional mountain villages the prevalence of asthma seems to have remained unchanged. The higher prevalence of atopic diseases in the more accessible coastal villages could be due to the rise of Western influence in recent decades.

### 4. Lifestyle Differences of Karkar Islanders Suffering from an Atopic Disease

Lifestyle differences of Karkar Islanders suffering from an atopic disease compared with nonatopic subjects suggest a link between modern lifestyle and atopic diseases as well. Islanders suffering from an atopic disease tended to work more often in typical Western occupations. Furthermore, they took significantly more acetylsalicylic acid. This does not mean that the drug itself causes atopic diseases, although nonsteroidal anti-inflammatory drugs can enhance histamine release,[23] but acetylsalicylic acid may be regarded as an indicator for a more modern lifestyle and increased Western contact. Just 1 person (excluded) indicated taking acetylsalicylic acid to relieve atopic symptoms. Therefore, the abovestated difference cannot be explained as a consequence of the treatment of atopic diseases with acetylsalicylic acid.

The idea that an increase in Western influence may also increase the prevalence of atopic diseases was already supported by Dowse, Turner, and co-workers in 1985. They showed a correlation between the increasing prevalence of asthma and the introduction of blankets in the Papua New Guinea highlands [24]. However, Western textiles are not likely to be the only trigger for the development of allergies and they cannot explain the higher prevalence of atopic diseases in the coastal villages of Karkar Island. Meanwhile, Western textiles are widespread in all villages on Karkar Island. Nevertheless, there seems to have been a significant increase in the prevalence of atopic diseases in the coastal villages where a multitude of different aspects of Western lifestyle have been introduced. There is also evidence from Western countries suggesting that retaining a more traditional lifestyle in the farmhouse (Bavaria, Germany)[25] or having an anthroposophic lifestyle (Steiner school children)[26] may be accom-panied by a lower incidence of atopic diseases.

## Conclusions

In summary, the results of our observations in the islanders of Papua New Guinea support the notion that a traditional lifestyle may protect against atopic diseases. The exact mechanisms and the most relevant single factors remain to be elucidated in future studies.

## End Notes

Supported by: Deutsche Gesellschaft für Allergologie und Klinische Immunologie, Germany.
